# Efficacy of Internal Heat Acupuncture Combined with High-Voltage Long-Duration Pulsed Radiofrequency on Subacute Postherpetic Neuralgia: A Retrospective Study

**DOI:** 10.1155/2022/2180214

**Published:** 2022-06-10

**Authors:** Yong Fei, Ming Yao, Bing Huang, Longsheng Xu, Beibei Liu

**Affiliations:** ^1^Department of Anesthesiology and Pain Medicine, Affiliated Hospital of Jiaxing University, Jiaxing 314001, Zhejiang, China; ^2^Department of Central Laboratory, Affiliated Hospital of Jiaxing University, Jiaxing 314001, Zhejiang, China

## Abstract

**Objectives:**

This study aims at investigating the internal heat acupuncture (IHA) combined with the high-voltage long-duration pulsed radiofrequency (PRF) therapeutic effect on subacute postherpetic neuralgia (PHN).

**Methods:**

This retrospective study comprised 81 cases with PHN. They were divided into three groups: IHA combined with the high-voltage long-duration PRF group (IHA-PRF), intradermal injection combined with the high-voltage long-duration PRF group (II-PRF), and the high-voltage long-duration PRF group. The pain numerical rating score (NRS), IL-6, Gal-3, and blood glucose levels were recorded before and after treatment.

**Results:**

Compared with before treatment, NRS scores of the three groups were all decreased at each time point. NRS scores of the IHA-PRF group patients decreased significantly in comparison to the PRF group at 1, 4, 8, and 12 weeks following treatment, while group II-PRF only decreased significantly at one week following treatment. Compared with groups II-PRF and PRF, respectively, IL-6 and Gal-3 levels in plasma of patients in group IHA-PRF were significantly decreased at 4 and 12 weeks after treatment. The effective rate of group IHA-PRF was 88.9%, which was considerably more than the other groups, II-PRF (63.0%) and PRF (63.0%). Compared with group II-PRF, patients' blood glucose levels in IHA-PRF and PRF groups significantly decreased three days and one week after treatment.

**Conclusion:**

Internal heat acupuncture combined with high-voltage long-duration pulsed radiofrequency has a satisfactory therapeutic effect on subacute PHN and has no obvious adverse reactions, which is especially suitable for patients with poor blood glucose control.

## 1. Introduction

Herpes zoster is caused by varicella zoster virus infecting the dorsal root ganglion or cranial nerve, which is more common in middle-aged and elderly people with reduced immunity [1]. Neuralgia is one of the most common and serious symptoms of herpes zoster, which seriously affects the life quality of patients and increases the medical burden [2, 3]. Nerve regulation technology is one of the effective methods for clinical treatment of PHN [4]. PRF technology is widely used in the treatment of PHN because of its simple operation, low cost, and high patient acceptance [5, 6]. However, the standard PRF therapy for PHN is often difficult to achieve the lasting therapeutic effect [7]. It was reported that high-voltage long-duration PRF is more effective than standard PRF in the treatment of PHN in acute and subacute stages [8]. However, most patients still have residual pain on the skin surface after operation. It was reported that the internal heat acupuncture therapy is effective, safe, and reliable in the treatment of poststroke shoulder pain [9].

Due to the complexity of pathological changes of PHN, some patients need to be combined with other treatment methods to relieve pain [10]. PHN is not only related to acute nerve injury but also closely related to local ischemia, neurotrophic disorder, and scar formation caused by arteriosclerosis of the supporting nerve [11]. Therefore, alleviating the local blood circulation in the lesion area is also one of the effective methods for the treatment of PHN. Therefore, this study proposes silver needle heating combined with radiofrequency therapy for the treatment of PHN.

In this study, internal heat acupuncture combined with high-voltage long-duration PRF were used to treat subacute stage PHN and observe the efficacy.

## 2. Materials and Methods

### 2.1. Participants

**Figure 1:**
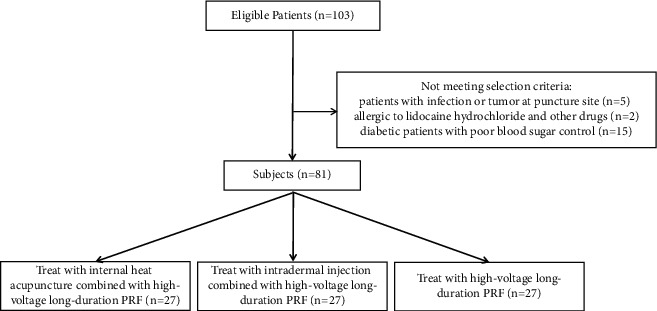
The study participants' flow diagram.

This research included patients on oral medication before presentation, and the degree of pain remained moderate to severe (NRS >3). There were 39 males and 42 females aged 44–86 years old, and the course of the disease ranged from 1 to 3 months. The nerve distribution area of all patients with PHN in the spinal segment includes 59 cases in thoracic segment and 22 cases in lumbar segment. This study comprised 115 cases with PHN from Pain Department, Affiliated Hospital of Jiaxing University. Patients with puncture site infection or tumor, allergic to drugs such as lidocaine hydrochloride and compound betamethasone, with severe cardiovascular and cerebrovascular diseases, abnormal bleeding and coagulation function, uncontrolled diabetes , liver and kidney dysfunction, serious mental illness, and inability to collaborate with surgery were excluded. After excluding these patients who do not meet the inclusion criteria, 81 patients were included in the study. According to the random number table method, they were divided into three groups: the IHA-PRF group (*n* = 27), the II-PRF group (*n* = 27), and the PRF group (*n* = 27). A summary of patient progression through the study is shown in Figure 1. All treatments were performed by the same senior pain doctor. The hospital ethics committee approved this research (approval number: LS2019-288). All subjects had informed consent to the research contents.

### 2.2. Therapeutic Methods

#### 2.2.1. PRF Therapeutic steps

**Figure 2:**
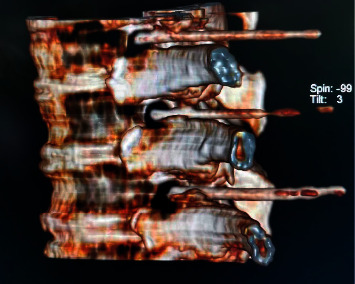
Three-position reconstruction of corresponding segments.

In the prone position, the patients were laid on a CT treatment bed, the most painful parts in the middle and one part extended up and down, respectively, with three segments each time to perform dorsal root ganglion's PRF therapy. The puncture site for CT placement was determined to be the ventral intervertebral foramen's upper edge, and the puncture pathway was developed. 1.0% lidocaine hydrochloride was used for local infiltrating anesthesia with a slow push of radiofrequency puncture (the parameters refer to our previous research [12]) under CT. Finally, the tip of the needle was located in the epigastric abdomen on the ventral side of the intervertebral foramen, and pain at the root from the site of the puncture to the chest wall may occur. RF testing is a sensory test that utilizes parameters used in our previous research [12]. Corresponding trunk muscle shake is observed in the inserted thoracic or lumbar segment. The three-position reconstruction diagram is shown in Figure 2. There was no blood, gas, or liquid in the puncture needle withdrawal, and 2 mL of 1.0% lidocaine hydrochloride is injected into each target for three minutes. Adjust the RF generator to manual pulse mode with temperature, time, pulse duration, pulse rate, and voltage set to 42°C, 900 s, 20 ms, 2 Hz, and 90 V, respectively. After RF was complete, remove the electrodes and needle, without blood, gas, or liquid, and inject 5 ml of mixed solution into each segment. The formula of the mixed solution included 2% lidocaine injection 100 mg (batch number H20133209, Tianjin Jinyao Pharmaceutical Co., Ltd.), 500 g of mecobalamin injection (batch number 190707A, Japan Eisai Co., Ltd.), compound betamethasone injection (batch number J20140160, Hangzhou Merck Pharmaceutical Co., Ltd.) 1 mL, recombinant human interferon-2b injection (batch number S20040010, Anhui Anke Biotechnology Engineering Co., Ltd.) 1,000,000 U, and 3 mL of 30% iodohyanol injection (batch number H20000591, Shanghai General Electric Pharmaceutical Co., Ltd.). Dilute the mixed solution with normal saline to 15 mL. After the needle was extracted, press at the puncture position. After the vital signs are stabilized, the patient is returned to the ward after 15 minutes of monitoring.

### 2.3. Internal Heat Acupuncture Treatment Steps

The skin lesion area was marked, the needle distance was designed to be 2 cm, and the internal heat-type acupuncture needle with a model of 0.70 mm ^*∗*^ 110 mm in the rear row entered the middle of the two needles in the front row with a needle distance of 1 cm. The needle entry method: after penetrating the subcutaneous tissue vertically, the angle between the needle and the skin was kept to 5°, and the internal heat acupuncture entered the subcutaneous tissue at 5 cm before being pushed forward slowly. According to this puncture method and design, the whole skin lesion area is covered. Generally, 10–20 needles were injected. A K-type internal heat acupuncture therapeutic apparatus (Shandong Jining Jiake Medical Technology Co., Ltd.) was connected, and the temperature was set at 42°C and heated for 20 min. Patients in group IHA-PRF were treated with internal heat acupuncture 1, 2, and 3 weeks following high-voltage, long-duration PRF operation.

### 2.4. Intradermal Injection Treatment Steps

Make circular punctate puncture around the painful part with the needle tip penetrating at an angle of 30–45 with the skin, slowly push the drug into the subcutaneous tissue, with a spacing of 5 cm at each point, and finally cover the whole skin lesion area with the injection and generally puncture 10–20 needles. Drug formula for intradermal injection: 2% lidocaine hydrochloride injection 5 ml, mecobalamin injection 0.5 mg, and compound betamethasone injection 1 ml, diluted with 0.9% sodium chloride injection to 20 ml. Patients in group II-PRF were treated with intradermal injection at 1, 2, and 3 weeks after high-voltage long-duration PRF operation.

### 2.5. Data Collection

The pain degree of patients was evaluated by the numerical rating scale (NRS): zero means painless and ten means unbearable pain. The patient's NRS scores prior to and following the treatment were assessed at three days, 1, 4, 8, and 12 weeks. The plasma IL-6 and Gal-3 levels were assessed prior to treatment and three days, 1, 4, and 12 weeks following treatment. To evaluate the curative effect, it was effective to reduce the NRS score by 25% or more than before treatment. Among them, a reduction of 80% or more is excellent, 40–80% is good, 25–40% is acceptable, and less than 25% is poor. The effective rate was recorded 12 weeks after treatment. The effective rate was the ratio of the number of cases whose NRS score decreased by 25% or more to the total number of cases.

### 2.6. Statistical Analysis

A normal distribution of measurement data was analyzed using the SPSS 21.0 statistical software. The mean standard deviation was used to express measurement data. In this study, repeated measurement data were analyzed by multivariate analysis, pairwise comparisons were carried out by the LSD test, and counting data were compared by the chi-square test. A *P* value of 0.05 was considered statistically significant.

## 3. Results

### 3.1. The Patients' General Data in Each Group Had No Significant Difference

Prior to presentation, all participants were on oral medication. The pain degree ranged from moderate to severe. No significant differences in gender, age, disease course, or nerve distribution area were observed (*P* > 0.05) (Table 1).

### 3.2. NRS Scores Decreased after Treatment in Each Group

**Figure 3:**
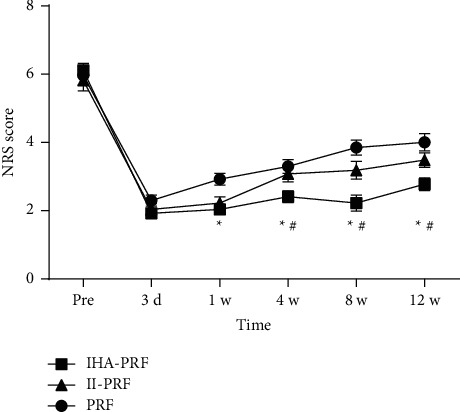
NRS scores decreased after treatment in each group. Compared with before treatment, NRS scores of the three groups decreased at each time point after treatment. NRS scores of patients in group IHA-PRF significantly decreased at 4, 8, and 12 weeks after the treatment course. NRS scores of patients in group IHA-PRF decreased at 1, 4, 8, and 12 weeks after the treatment course. NRS scores of patients in group II-PRF decreased one week after the treatment course. Compared with group II-PRF, ^*∗*^*P* < 0.05. ^#^*P* < 0.05, compared with group PRF.

**Figure 4:**
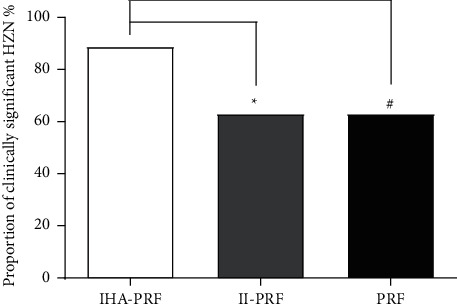
The group IHA-PRF effective rate was considerably higher than the other two groups. Patients in the three groups were followed up for 12 weeks following treatment. The effective rate in group IHA-PRF (88.9%) was considerably higher than group II-PRF (63.0%) and group PRF (63.0%). Compared with group II-PRF, ^*∗*^*P* < 0.05. ^#^*P* < 0.05, compared with group PRF.

Compared with pretreatment, all three groups of patients had decreased their NRS scores at various points in time after treatment. In comparison to group II-PRF, the patients' NRS scores in group IHA-PRF decreased significantly at 4, 8, and 12 weeks following the treatment course (*P* < 0.05). In comparison to group PRF, the patients' NRS scores in group IHA-PRF decreased at 1, 4, 8, and 12 weeks after the treatment course. NRS scores in the II-PRF group decreased significantly compared to the PRF group after 1 week of treatment (*P* < 0.05), as shown in Figure 3. The three groups were followed up for 12 weeks following treatment. The effective rate of group IHA-PRF was 88.9%, which was considerably more than the other groups, II-PRF (63.0%) and PRF (63.0%), with a statistical difference (*P* < 0.05) (Figure 4). Complications such as local anesthetic poisoning, nausea and vomiting, and infection at the treatment site were not found in all three groups.

### 3.3. The Amount of Gal-3 and IL-6 in Plasma Decreased following Treatment in Group IHA-PRF

**Figure 5:**
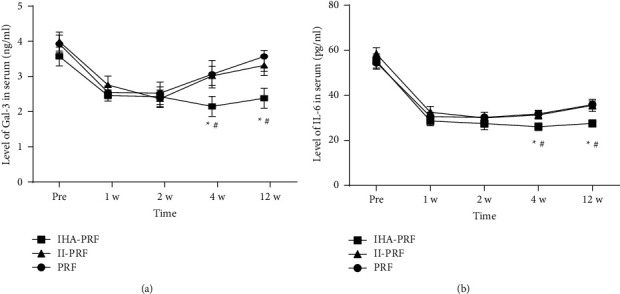
Following treatment in group IHA-PRF, plasma amount of Gal-3 (a) and IL-6 (b) decreased in each group. Compared to II-PRF and PRF groups, the amount of Gal-3 and IL-6 in plasma of patients in group IHA-PRF significantly decreased 4 and 12 weeks after treatment. Compared with group II-PRF, ^*∗*^*P* < 0.05. ^#^*P* < 0.05, compared with group PRF.

Following treatment, the amount of Gal-3 and IL-6 in plasma of each group decreased in comparison to prior treatment. Compared to II-PRF and PRF groups, the amount of Gal-3 and IL-6 in plasma of patients in group IHA-PRF decreased, respectively, four and twelve weeks after treatment (*P* < 0.05) (Figure 5).

### 3.4. The Patients' Blood Glucose Levels in IHA-PRF and PRF Groups Decreased Three Days and One Week after Treatment

**Figure 6:**
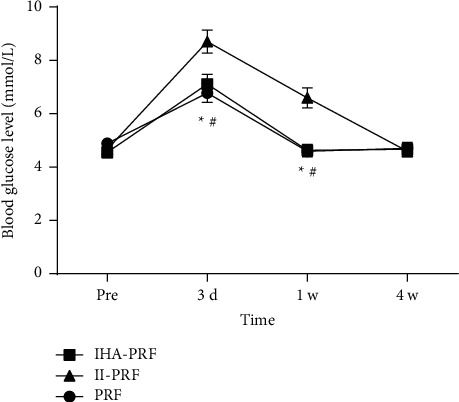
The blood glucose levels of patients in group IHA-PRF and group PRF decreased three days and one week after treatment. Compared with before treatment, the patients' blood glucose in IHA-PRF and PRF groups increased three days after the treatment course and that of patients in group II-PRF increased three days and one week after the treatment course with significant differences. Patients' blood glucose levels in IHA-PRF and PRF groups decreased three days and one week after treatment. Compared with group II-PRF, ^*∗*^*P* < 0.05. ^#^*P* < 0.05, compared with group PRF.

Compared with before treatment, the patients' blood glucose in group IHA-PRF and group PRF increased three days after the treatment course and that of patients in group II-PRF increased three days and one week after the treatment course with significant differences (*P* < 0.05). In comparison to group II-PRF, the patients' blood glucose levels in IHA-PRF and PRF groups decreased significantly three days and one week following treatment (*P* < 0.05) (Figure 6).

## 4. Discussion

PHN is difficult to be treated clinically because of its complicated pathogenesis [13]. At present, all clinical treatments are aimed at reducing PHN occurrence. Due to the lack of understanding of pain departments, many patients with shingles neuralgia have mostly come to the pain department in the subacute phase (1-3 months of course) and even have complicated postherpetic neuralgia. The pathological features of PHN in the subacute stage are that skin lesions have scabbed with severe neuralgia [14]. These patients are extremely prone to complications with PHN and require timely treatment. PRF of dorsal root ganglion is an intermittent pulsed current emitted by a radio generator, which acts around the diseased dorsal root ganglion and nerve fibers [15]. It plays an analgesic role by recuperating the disordered electrical signals and reducing the substance P level of the dorsal root ganglion [16]. Since the maximum temperature of the electrode tip at the time of treatment is 42°C, the lower temperature does not destroy the physiological basis of nerve impulse transmission, let alone motor nerve function. Compared with traditional continuous radiofrequency, it has no thermal damage to the nerve and will not aggravate the original neuropathic pain. However, its action intensity is limited due to the low field strength (40 V) and short duration (180–300 s) of standard voltage PRF treatment. It cannot make patients get a lasting treatment effect and cannot significantly reduce PHN incidence. Teixeira et al. found that PRF field strength was positively correlated with the therapeutic effect [17]. Wan C et al. reported that high-pressure, long-acting PRF can be applied to the treatment of PHN [8]. The field strength was manually adjusted and gradually increased according to the patient's tolerance (maximum 90 V). The treatment duration was 900 s. NRS of patients with PHN decreased at 1, 4, 8, and 12 weeks after operation, and the quality of life improved. However, most patients still have residual pain on the skin surface after operation [18]. Some scholars have reported that internal heat acupuncture can solve the pain of nerve endings on the herpes zoster skin surface and improve the life quality and patients' satisfaction [19].

In this study, compared with before treatment, the NRS score of patients treated with high-voltage long-duration PRF of the dorsal root ganglion combined with internal heat acupuncture decreased significantly after treatment, indicating that the curative effect of internal heat acupuncture was more lasting than that of intradermal injection. The mechanism may eliminate inflammation of skin nerve endings and release local muscles.

Some scholars have studied that after varicella zoster virus infection, the expression of Gal-3 in the mice spinal cord's dorsal horn increased significantly, and the intrathecal injection of Gal-3 antibody in mice with Gal-3 gene deletion significantly reduced the touch-induced pain, indicating that Gal-3 was involved in PHN production [20]. Additionally, Gal-3 may mediate PHN through macrophages and microglia. Plasma Gal-3 level and the neuropathic pain severity had a positive correlation [21, 22]. IL-6 can promote the growth and differentiation of primitive bone marrow source cells and has the function of enhancing the immune response. IL-6 has a pivotal role in the antiinfection immune response. The amount of IL-6 and nerve injury had a positive correlation. A high IL-6 level is the main factor that leads to nerve injury and chronic pain development and may have a pivotal role in neuropathic pain development [23]. Our previous research results also revealed that in PHN patients, Gal-3 and IL-6 plasma levels were considerably higher than in normal people [12]. It can be used as an early diagnosis and a predictor of PHN [24]. In this study, it was found that Gal-3 and IL-6 levels in the patients' plasma in the group of internal heat acupuncture combined with high-voltage long-duration PRF decreased significantly at 4 and 12 weeks after treatment, implying that the effect of internal heat acupuncture on eliminating skin nerve endings inflammation was more lasting.

Complications such as local anesthetic poisoning, nausea, vomiting, and infection at the treatment site were not found in all three groups. On the 3^rd^ day after treatment, patients' blood glucose in the three groups increased. This study found that the blood glucose level of patients in IHA-PRF and PRF groups decreased more significantly than in group II-PRF three days and one week after treatment, demonstrating that internal heat acupuncture combined with high-voltage long-duration PRF treatment is more suitable for patients with poor blood glucose control.

This research faced some limitations. First, there may be differences between the actual pain score and the expressed value. Second, the sample size is relatively small, and there is a short follow-up time. Further long-duration observation is required for determining the duration of internal heat acupuncture combined with the high-voltage long-duration PRF effect on subacute PHN. To verify our results in a larger population, larger sample size and an appropriate control group will be required to overcome the limitation of a small sample size.

## 5. Conclusion

In summary, treating subacute PHN with internal heat acupuncture combined with high-voltage long-duration PRF of the dorsal root ganglion has a satisfactory therapeutic effect and no obvious adverse reactions, which is especially suitable for patients with poor blood glucose control.

## Figures and Tables

**Table 1:** General data of patients.

	Number of cases	Age (years)	Gender (male/female)	Course of disease (months)	Chest/waist segment (example)
Group IHA-PRF	27	66.19 ± 10.792	13/14	1.74 ± 0.656	21/6

Group II-PRF	27	64.70 ± 10.894	12/15	1.74 ± 0.526	18/9

Group PRF	27	66.15 ± 11.055	14/13	1.78 ± 0.801	20/7

*t*/*χ*^2^ value		0.161	0.297^a^	0.027	0.847^a^

*P* value		0.852	0.862	0.973	0.646

^a^The value of *χ*^2^.

## Data Availability

The data and materials that support the findings of this study are available from the corresponding author upon reasonable request.
